# Prevalence and Associated Risk Factors of Indigestible Foreign Bodies in the Forestomach of Slaughtered Cattle: A Cross‐Sectional Study in Eastern Ethiopia

**DOI:** 10.1155/vmi/1616641

**Published:** 2026-09-16

**Authors:** Andnet Yirga Assefa, Mekoya Tefera Mamo, Temesgen Sendekie Ayalew

**Affiliations:** ^1^ Department of Veterinary Epidemiology and Public Health, School of Veterinary Medicine, Bahir Dar University, Bahir Dar, Ethiopia, bdu.edu.et

**Keywords:** abattoir, cattle, foreign bodies, forestomach, Haramaya, prevalence, risk factors

## Abstract

Indigestible foreign bodies (IFBs) can impair digestion and reduce animal productivity, thereby posing a serious economic burden on livestock owners and the national economy. A cross‐sectional study was conducted from November 2024 to March 2025 to assess the prevalence and associated risk factors of IFBs in the forestomach of cattle slaughtered at Haramaya University and Haramaya Municipal Abattoirs, Oromia region, Eastern Ethiopia. A total of 384 cattle were selected randomly and examined postmortem for the presence of IFBs. The overall prevalence of IFBs in cattle was 35.7% (137/384; 95% confidence interval [CI]: 31.1%–40.6%). The most important risk factors significantly associated with the occurrence of IFBs were poor body condition (adjusted odds ratio [AOR] = 3.7; 95% CI: 1.72–8.0), older age (AOR = 4.8; 95% CI: 2.41–9.97), being from rural areas (AOR = 2.2; CI: 1.15–4.39), and being female (AOR = 3.8; 95% CI: 2.10–7.03), while breed of cattle showed no significant association. Among the forestomach compartments, a higher prevalence of foreign bodies was detected in the rumen (67.15%) than in the reticulum (32.85%). Nonmetallic materials accounted for the majority of detected IFBs (80.3%), with plastic objects being the most common (37%). The high prevalence observed in this study underscores the widespread environmental contamination and inadequate solid waste disposal practices in the area. Therefore, public awareness and the implementation of proper waste management systems are needed to minimize the ingestion of foreign materials by cattle and improve animal health and production.

## 1. Introduction

Livestock is an important agricultural sector of Ethiopia, as almost all farmers own a variable number of livestock [1]. According to the livestock sample survey, Ethiopia has a cattle population of over 70.3 million, more cows than any other African country [2, 3]. However, the marketing system for live animals and meat remains underdeveloped due to several challenges that impede efficient transactions, including poor infrastructure and traditional production methods [4].

In Ethiopia, cattle are predominantly raised under extensive and mixed crop–livestock production systems [5], where animals depend mainly on communal grazing lands, natural pastures, crop residues, and, to a lesser extent, agro‐industrial by‐products. Seasonal feed shortages, expansion of urban settlements, and increasing cultivation of cash crops have reduced available grazing land [6], forcing cattle to graze along roadsides, around marketplaces, and near open waste disposal sites. Consequently, cattle are at an increased risk of ingesting indigestible materials such as plastic bags, cloth, ropes, and metallic objects while searching for feed [7, 8, 9].

Sustainable livestock production is a critical component of global food security and environmental stewardship [10]. However, both infectious and noninfectious diseases continue to compromise livestock productivity and welfare [11]. Among these conditions, ingestion of indigestible foreign bodies (IFBs) has emerged as an important health problem in cattle worldwide, particularly in areas where feed resources are limited and grazing lands are contaminated with solid waste [7, 12].

Most of the nonmetallic foreign bodies are lodged in the rumen, while metallic foreign bodies are lodged in the reticulum [13]. The pathogenesis of IFB‐related diseases begins with consuming solid waste material (SWM) [11, 14]. This, in turn, results in clinical conditions such as inflammation of the oral and digestive mucosa, ruminal impaction, traumatic reticuloperitonitis (TRP), and pericarditis [15], and death following intestinal tract obstruction [16]. These conditions arise from sharp objects, like metal fragments, piercing the reticulum and potentially spreading infection or causing inflammation in surrounding tissues [15, 17].

The problem of waste disposal has become “endemic” to tropical environments, with adverse effects exerted on both human and animal populations [18, 19]. These diseases have a deleterious economic impact on smallholder farmers, resulting in losses due to decreased productivity, treatment costs, mortality rates, and even loss of desire for ruminant farming [20–23]. Moreover, certain plastic materials may release chemical additives, such as phthalates and bisphenol compounds, during degradation, while plastics may also adsorb environmental contaminants. Although the extent to which these substances transfer into edible animal tissues remains under investigation, ingestion of plastic waste is recognized as a potential animal and public health concern because of its possible contribution to chemical exposure through meat and milk consumption [24].

Among several health issues affecting cattle in Ethiopia, the ingestion of indigestible foreign materials is a notable concern [7, 23, 25–27]. It is a problem for individual farmers in particular and Ethiopia in general [28]. Among the few studies conducted in Ethiopia, indigestible rumen and reticulum foreign bodies of selected districts of East Hararge Zone municipal abattoirs indicate ingestion of foreign bodies, particularly plastic materials, rope, and cloth, by animals [7].

Despite this issue being of considerable importance, there is a notable lack of comprehensive data in Ethiopia about the occurrence and magnitude of IFBs in the forestomach of domestic ruminants and their associated risk factors. Furthermore, there is insufficient information on the nature, anatomical location, and type of foreign bodies affecting cattle. Haramaya University and Haramaya Municipal abattoirs, both located in Haramaya town, Eastern Ethiopia, routinely slaughter cattle originating from Haramaya and neighboring areas, including Gara Mulata, Chalanko, Hirna, Debaso, Kurfa Chelle, Awaday, Kombolcha, Adele, and Away. However, the burden of IFBs in animals slaughtered in those abattoirs remains undocumented, reflecting a substantial gap in the available epidemiological evidence. Therefore, this study was conducted to estimate the prevalence of IFBs and their associated risk factors in the forestomach of cattle slaughtered at Haramaya University and Haramaya municipal abattoirs.

## 2. Materials and Methods

### 2.1. Study Area

**FIGURE 1:**
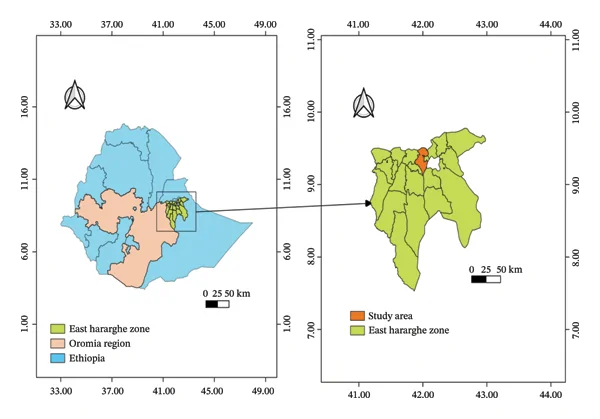
Map of the study area.

The study was conducted at Haramaya Municipal and Haramaya University Abattoirs in Haramaya, Eastern Hararghe Zone, Oromia Regional State, Eastern Ethiopia (Figure 1). Haramaya town is situated about 580 km east of Addis Ababa, the capital city of Ethiopia, and 14 km west of Harar. The town is also close to Lake Haramaya, a seasonal freshwater lake. It is known for various types of agricultural practices like crop production, cultivation of khat, and livestock production. Its elevation ranges from 1600 to 2100 m above sea level between 9° 24′N latitude and 42° 0′E longitude. Rainfall was heavy in the region, mostly from June to September, which is the main rainy season. The rainy season, which lasted from February to May, was also shorter. The average maximum and minimum temperatures are 17 and 9, respectively, and the mean annual rainfall is 870 mm with a range of 560–1260 mm annually. There are 64,510 cattle in the Haramaya district [29].

The Haramaya University abattoir is situated within the main campus of Haramaya University and is exclusively dedicated to the slaughter of cattle. It has a daily processing capacity of approximately 10–15 animals. In contrast, the Haramaya Municipal abattoir is located in Maya, within Haramaya town, and serves the surrounding community by providing slaughter services for a variety of livestock species, including cattle, sheep, goats, and camels. This facility processes between 30 and 40 cattle per day, in addition to other animal species.

### 2.2. Study Population

The study population consisted of apparently healthy cattle of different ages, sexes, breeds, origins, and body condition groups purchased from local marketplaces around Haramaya and presented for slaughter at Haramaya University and Haramaya Municipal abattoirs. The age of each animal was estimated based on dental eruption patterns, as described by Pace and Wakeman [30]. Cattle were categorized into three age groups: young (less than 5 years), adult (5 to 10 years), and old (above 10 years).

The body condition status of sampled animals was estimated using the method described by Parsons [31], which categorizes animals into nine body condition scores. Therefore, based on this technique, the animals were categorized into three groups as poor (scores 1–3), medium (scores 4–6), and good (scores 7–9) based on the level of muscling and fat deposition over and around the spines and transverse processes.

Based on the area of origin, cattle were categorized as urban, peri‐urban, or rural. Urban cattle were those originating from towns with predominantly intensive or market‐oriented production systems. Peri‐urban cattle originated from areas surrounding urban centers where mixed crop‐livestock and semi‐intensive production systems are common. Rural cattle were those originating from villages where livestock are primarily managed under extensive grazing systems. The major areas of origin included Gara Mulata, Chalanko, Hirna, Debaso, Kurfa Chelle, Awaday, Kombolcha, and Adele.

### 2.3. Study Design

A cross‐sectional study was conducted from November 2024 to March 2025 in Haramaya municipal and Haramaya University abattoirs to determine the prevalence of IFBs in the forestomach and to identify their associated risk factors in cattle slaughtered in both abattoirs.

### 2.4. Sampling Methods

Cattle were selected using a simple random sampling technique based on the daily slaughter register at each abattoir. All cattle presented for slaughter were listed in the daily slaughter register and assigned a unique identification number. The register served as the sampling frame, and the required number of animals was selected using a random number table. If a selected animal was excluded before slaughter (due to illness or removal from the slaughter line), another animal was randomly selected from the remaining eligible cattle. This procedure was repeated daily until the required sample size of 384 cattle was obtained.

### 2.5. Sample Size Determination

The sample size was calculated according to the formula given by Thrusfield [32] with a 95% confidence interval (CI) and 5% absolute precision. Since no previous study had been conducted in the study area, a 50% expected prevalence was used. Accordingly, the total sample size was determined to be 384.

(1) N=Z2×Pexp1−PexpD2,

(2) Hence, N=1.962×0.510.5−0.052=384,

where *N* = sample size, *P*_exp_ = expected prevalence (50%), *Z* = 1.96 (the value of *Z* at 95% CI), and *D* = desired absolute precision.

### 2.6. Data Collection Method

#### 2.6.1. Ante‐Mortem Examination

Ante‐mortem inspection was conducted by applying a specific range of procedures that consider the behavior and appearance, as well as signs of disease in animals [33]. Regular visits were conducted for the animals brought for slaughter. Each individual animal was assessed, and a unique identification code was assigned, and its place of origin, age, sex, breed, and body condition were recorded. The examinations were carried out under adequately illuminated conditions where the cattle were examined both at rest and during locomotion in the lairage, with the objective of determining their overall health status by assessing behavior, body condition, hygiene or cleanliness, signs of illness, and various abnormalities in respiration, body temperature, heart rate, locomotion, posture, anatomical structure, and conformation. Also any exudate arising from any bodily opening and its coloration were observed and recorded. Cattle exhibiting signs of optimal health and having received adequate rest were approved as fit for slaughter.

#### 2.6.2. Postmortem Examination

During the postmortem examination, the stomach was removed after dressing and opening the abdominal cavity. The rumen, reticulum, omasum, and abomasum were examined through visualization, incision, and palpation. When foreign bodies were encountered, they were removed, washed, identified, recorded, and photographed when possible.

### 2.7. Data Management and Analysis

The collected raw data were entered into a Microsoft Excel spreadsheet, coded, and screened for errors and missing data. Data analysis was conducted using STATA statistical software version 14 (Stata Corp, TX, USA). Descriptive statistics were employed to summarize the characteristics of the study population and the types and anatomical locations of IFBs. The overall prevalence of IFBs was estimated and presented with 95% CIs (95% CI). Univariable logistic regression was utilized to assess the association between each independent variable (sex, age, origins, body condition scores, and breed) and the presence of foreign bodies. Variables with a *p* value < 0.25 were considered for multivariable analysis. Multivariable logistic regression was then used to evaluate the combined effects of these variables on the presence of IFBs. The results are presented as crude odds ratios (CORs) and adjusted odds ratios (AOR) with corresponding 95% CIs. All statistical tests were two‐sided, and statistical significance was determined a priori at *p* < 0.05.

## 3. Results

### 3.1. Overall Prevalence

Out of 384 cattle examined, 137 (35.7%; 95% CI: 31.1%–40.6%) were found to harbor various types of foreign bodies in their forestomach, mainly in the rumen and reticulum.

### 3.2. Risk Factors Associated With the Occurrences of IFBs

Results of the univariable logistic regression are presented in Table 1. All the variables, including sex, age, body condition, breed, and origin of animals, were selected for the multivariable model (*p* < 0.25). The final model showed being female (AOR = 3.8; 95% CI = 2.10–7.03; *p* < 0.001), old age (AOR = 4.8; 95% CI = 2.41–9.97; *p* < 0.001), poor body condition (AOR = 3.7; 95% CI = 1.72–8.0; *p* < 0.001), and originating from a rural area (AOR = 2.2; 95% CI = 1.15–4.39; *p* = 0.02) as significant factors associated with the occurrence of IFBs (Table 1).

**TABLE 1:** Logistic regression analysis of the factors associated with the occurrence of indigestible foreign bodies in cattle.

Variables	Category	Not examined	No. of positive (%)	Univariable		Multivariable	
				COR (95% CI)	*p* value	AOR (95% CI)	*p* value
Sex	Male	312	92 (29.49)	Ref	Ref	Ref	Ref
	Female	72	45 (62.5)	4 (2.34–6.89)	< 0.001	3.8 (2.10–7.03)	< 0.001

Age	Young	127	29 (22.83)	Ref	Ref	Ref	Ref
	Adult	195	67 (34.36)	1.8 (1.07–2.97)	0.03	1.4 (0.79–2.46)	0.25
	Old	62	41 (66.13)	6.6 (3.42–13.07)	< 0.001	4.8 (2.41–9.97)	< 0.001

Body condition	Good	154	42 (27.27)	Ref	Ref	Ref	Ref
	Medium	187	68 (36.36)	1.5 (0.96–2.44)	0.07	1.6 (0.95–2.66)	0.08
	Poor	43	27 (62.79)	4.5 (2.23–9.39)	< 0.001	3.7 (1.72–8.0)	< 0.001

Breed	Local	355	122 (34.37)	Ref	Ref	Ref	Ref
	Exotic	29	15 (51.72)	2 (0.95–4.44)	0.06	1.8 (0.76–4.31)	0.18

Origin	Urban	76	21 (27.63)	Ref	Ref	Ref	Ref
	Peri‐urban	122	40 (32.79)	1.3 (0.68–2.44)	0.44	1.4 (0.70–2.89)	0.35
	Rural	186	76 (40.86)	1.8 (1.02–3.29)	0.04	2.2 (1.15–4.39)	0.02

*Note:* No, number.

Abbreviations: AOR, adjusted odds ratio; CI, confidence interval; COR, crude odds ratio.

### 3.3. Types and Frequency of IFBs in Forestomach of Cattle

Regarding the types of foreign bodies, 110 (80.3%) were nonmetallic, and 27 (19.71%) were metallic. The most common nonmetallic items were plastics (27%), stones (16.06%), and clothes (12.41%), while wires (8.03%), nails (5.11%), and needles (3.65%) were the most frequently detected metallic objects (Table 2).

**TABLE 2:** Types and frequency of indigestible foreign bodies in the forestomach of cattle.

Types of indigestible foreign bodies	Frequency (%)	Total (%)
Metallic foreign bodies		
Wire	11 (8.03)	27 (19.71)
Nail	7 (5.11)	
Needle	5 (3.65)	
key	1 (0.73)	
Blade	3 (2.19)	
Nonmetallic foreign bodies		
Plastics	37 (27.00)	110 (80.29)
Clothes	17 (12.41)	
Ropes	15 (10.95)	
Plastics and ropes	3 (2.19)	
Plastic and clothes	7 (5.11)	
Clothes and rope	2 (1.46)	
Leather	7 (5.11)	
Stones	22 (16.06)	
Total	137 (35.7)	

### 3.4. Location of IFBs in Forestomach of Cattle

From the positive cases (*N* = 137), 94 (68.61%) and 43 (31.39%) were found in the rumen and reticulum, respectively (Table 3).

**TABLE 3:** Location of indigestible foreign bodies in the forestomach of cattle.

Location	Identified foreign bodies		Frequency (%)
	Metallic foreign bodies	Nonmetallic foreign bodies	
Rumen	3	91	94 (68.61)
Reticulum	24	19	43 (31.39)

## 4. Discussion

Cattle’s ingestion of indigestible foreign objects is a widespread issue around the world [34, 35], one of the most significant diseases that necessitate surgical intervention [21]. The present study revealed an overall prevalence of 35.7% (95% CI: 31.1%–40.6%) of IFBs in the forestomach, mainly rumen and reticulum, of cattle slaughtered at Haramaya University and Haramaya municipal abattoirs.

The findings of the current study were in agreement with the report of Bitew [25] and Kebede et al. [35], who reported a prevalence of 36.5% and 37.5% in cattle slaughtered at Gondar Elfora abattoir and Kombolcha Elfora abattoir, respectively. However, this result was higher than the reports of Nebebe [36], Mulatu et al. [37], Jebessa et al. [38], Bihon et al. [39], Abera et al. [13], and Duresa et al. [19] in different parts of Ethiopia. The higher prevalence of foreign bodies in the current study area is most likely associated with the improper disposal of plastic bags and other indigestible materials, as well as their widespread use in the study area [22]. The study area and the nearby areas are largely covered by a plant called Khat (*Catha edulis*). Expansion of khat cultivation has progressively reduced communal grazing land, forcing cattle to graze on roadsides, waste disposal sites, and degraded areas where indigestible materials accumulate.

The finding of the current study is lower than the prevalence reported by Anwar et al. [40], Sheferaw et al. [41], Nongcula et al. [42], and Ame et al. [21]. These variations in prevalence could be driven by variations in the degree of foreign body contamination in the environment and/or in the management and husbandry practices used in the various study areas, which would impact the availability of IFBs or the cattle’s access to such items [19]. Factors like waste disposal systems in the areas where cattle graze, pollution levels, the likelihood that the cattle will be exposed to foreign bodies, and the use of plastic bags for carrying may also contribute to these differences [38]. Furthermore, during feed shortages, animals in Ethiopia consume unusual materials like plastics, clothing, ropes, and metals, especially during the dry season from December to early March [13].

The prevalence of IFBs was significantly higher in female cattle (62.5%) compared to male cattle (29.5%). Female cattle were 3.8 times more likely to harbor IFBs than their male counterparts (AOR = 3.8). This finding is consistent with previous reports by Mulatu et al. [37], Kebede et al. [35], and Adoko [43], all of whom documented a higher prevalence of IFBs in female cattle.

The sex disparity may be attributed to increased feed intake among female animals, particularly during physiologically demanding periods such as pregnancy and lactation, when nutritional requirements are elevated, and feed shortages may lead to the ingestion of nonnutritive materials [38]. Additionally, the greater demand for minerals such as calcium and phosphorus during late gestation and early lactation, essential for fetal development and milk production, may contribute to this behavior [44]. Furthermore, female cattle are often retained longer in herds for milk production and reproduction, which may increase their cumulative exposure to indigestible foreign materials over time [37].

The present study revealed the highest prevalence of IFBs in older cattle (66.13%), followed by adults (34.36%) and young cattle (22.83%). Compared to young cattle, older and adult cattle were 4.8 times and 1.4 times more likely, respectively, to harbor IFBs (AOR = 4.8 and AOR = 1.4). This finding is consistent with previous reports by Desiye and Mersha [45], Nebebe [36], Ame et al. [21], and Bitew [25], all of whom observed a higher frequency of foreign bodies in older cattle. The increased prevalence in older animals may be attributed to the gradual accumulation of indigestible materials ingested over time throughout their lifespan.

This study revealed a statistically significant association between body condition and the prevalence of IFBs in cattle. The highest prevalence was observed in animals with poor body condition (62.79%), followed by those with medium (36.36%) and good (27.27%) body condition. Cattle exhibiting poor and medium body conditions were 3.7 and 1.6 times more likely, respectively, to harbor IFBs compared to those in good condition. These findings are consistent with previous reports by Desiye and Mersha [45], Bassa and Tesfaye [46], Ali and Awoke [34], and Mekonnen et al. [22], who also documented a higher occurrence of IFBs in cattle with compromised body condition.

The higher prevalence of IFBs in cattle with poor body condition might be due to the negative impact of these materials on nutrient utilization, leading to progressive weight loss [47]. This could result from the retention of nondigestible objects in the forestomach, which disrupts the normal passage of ingesta and impairs the absorption of essential nutrients, particularly minerals and volatile fatty acids (VFAs) [48]. Consequently, such interference compromises metabolic efficiency and delays weight gain or fattening in affected animals [34, 49].

The current study showed that there is a statistically significant association between the origin of cattle and the occurrence of IFBs. A higher prevalence of IFBs was observed in cattle from rural areas (40.86%) compared to those from peri‐urban (32.79%) and urban (27.63%) areas. Cattle originating from rural and peri‐urban areas were 2.2 and 1.4 times more likely, respectively, to harbor IFBs than those from urban areas. This result contrasts with the findings of Mulatu et al. [37], who reported a higher prevalence of IFBs in cattle from urban areas (30.5%) compared to rural areas (7.8%) among cattle slaughtered in Hawassa.

This variation in IFB prevalence is more likely attributable to differences in waste management and livestock husbandry practices. In rural settings, improper disposal of waste materials on open grazing lands [50] and improper animal management practices [26] may contribute to increased exposure. The highest prevalence in rural areas may be explained by extensive grazing systems that bring animals into contact with poorly maintained environments containing plastic bags, ropes, and other indigestible items [35]. Although urban and peri‐urban environments also contribute to IFB occurrence, the relatively lower prevalence in these areas may be due to more controlled feeding practices and limited grazing opportunities, which reduce exposure to environmental contaminants [51, 52].

Although the association was not statistically significant, a higher proportion of IFBs was observed in crossbred cattle (51.72%) compared to local breeds (34.37%). This finding aligns with the reports of Rahel [53], Desiye and Mersha [45], Ame et al. [21], and Adoko [43], who also documented elevated rates in crossbred animals across various regions. The increased occurrence in crossbreeds may be linked to their greater body size, productivity, and draught capacity, which result in higher nutritional demands and feed intake. This elevated consumption potentially raises the likelihood of ingesting nonnutritive materials [54].

A higher proportion of IFBs was identified in the rumen (67.15%) compared to the reticulum (32.85%). This observation is consistent with previous findings by Remi‐Adewunmi et al. [48], Nebebe [36], Gurara et al. [55], and Nongcula et al. [14], who also reported a greater prevalence in the rumen. Conversely, this result contrasts with the findings of Kebede et al. [35], who recorded a higher concentration of foreign bodies in the reticulum. The predominance in the rumen might be due to its role as the primary and largest compartment of the forestomach, functioning as a major site for ingested material storage [56]. Nonmetallic objects, in particular, are more likely to become lodged in the rumen due to its capacity and slower ingesta turnover [57].

The prevalence of nonmetallic IFBs (80.3%) was higher compared to metallic materials (19.7%). Among the nonmetallic types, plastic materials were the most frequently encountered, accounting for 27% of the cases. This finding is consistent with previous reports by Abebe and Nuru [58] at Luna Export Abattoir; Remi‐Adewunmi et al. [48] in Zaria; Desiye and Mersha [45] from Jimma Municipal Abattoir; and Bihon et al. [39] and Bitew [25] from Gondar Elfora Abattoir. The high occurrence of plastic materials may be attributed to the widespread availability of plastic bags, ineffective waste disposal systems, limited public awareness regarding the harmful effects of plastic ingestion on livestock health and productivity, and improper management of plastic waste [39].

## 5. Conclusion

This study demonstrated that IFBs are a common problem among cattle slaughtered at Haramaya University and Haramaya Municipal abattoirs, with plastic bags being the most frequently recovered foreign body and the rumen the predominant site of accumulation. The occurrence of IFBs was significantly associated with age, body condition score, origin, and sex, with older cattle, animals in poor body condition, cattle originating from rural areas, and female cattle being at higher risk. These findings highlight the need for improved solid waste management, enhanced environmental sanitation, and better feed resource management to reduce cattle exposure to indigestible materials. Community awareness of proper waste disposal and sustainable livestock management practices should also be strengthened to minimize the health and economic impacts of foreign body ingestion. Further farm‐based studies incorporating detailed production and management factors are recommended to better understand the determinants of IFB ingestion in cattle.

## Funding

No funding was received for this research.

## Conflicts of Interest

The authors declare no conflicts of interest.

## Supporting Information

Additional supporting information can be found online in the Supporting Information section.

## Supporting information


**Supporting Information** Representative images of the major indigestible foreign bodies identified during the study are provided in the supporting information (Supporting figure 1).

## Data Availability

The data that support the findings of this study are available from the corresponding author upon reasonable request.
