# Supplementary material for: Prevalence and Associated Risk Factors of Indigestible Foreign Bodies in the Forestomach of Slaughtered Cattle: A Cross‐Sectional Study in Eastern Ethiopia
Source: Vet Med Int. 2026 Sep 16;2026:1616641. doi: 10.1155/vmi/1616641 (PMC13579382; doi:10.1155/vmi/1616641)
Supplement: Supplementary file 1 [file VMI-2026-1616641-s001.docx]

**Supplementary figure 1**: Figures showing indigestible foreign bodies extracted from slaughtered animals

Plastic and cloth	Leather	Stone

Cloth	Rope	Plastic and rope

Cloth	Leather
